# Analgesic Effects of Repetitive Transcranial Magnetic Stimulation in Patients With Advanced Non-Small-Cell Lung Cancer: A Randomized, Sham-Controlled, Pilot Study

**DOI:** 10.3389/fonc.2022.840855

**Published:** 2022-03-17

**Authors:** Ying Tang, Han Chen, Yi Zhou, Ming-liang Tan, Shuang-long Xiong, Yan Li, Xiao-hui Ji, Yong-sheng Li

**Affiliations:** ^1^ Chongqing Key Laboratory of Translational Research for Cancer Metastasis and Individualized Treatment, Chongqing University Cancer Hospital, Chongqing, China; ^2^ Department of Rehabilitation, Southwest Hospital, Army Medical University, Chongqing, China

**Keywords:** cancer pain, non-small-cell lung cancer, repetitive transcranial magnetic stimulation, quality of life, analgesic effects

## Abstract

**Objective:**

Current pharmacological intervention for the cancer-related pain is still limited. The aim of this study was to explore whether repetitive transcranial magnetic stimulation (rTMS) could be an effective adjuvant therapy to reduce pain in patients with advanced non-small cell lung cancer (NSCLC).

**Methods:**

This was a randomized, sham–controlled study. A total of 41 advanced NSCLC patients with uncontrolled pain (score≥4 on pain intensity assessed with an 11-point numeric rating scale) were randomized to receive active (10 Hz, 2000 stimuli) (n = 20) or sham rTMS (n = 20) for 3 weeks. Pain was the primary outcome and was assessed with the Numeric Rating Scale (NRS). Secondary outcomes were oral morphine equivalent (OME) daily dose, quality of life (WHO Quality of Life-BREF), and psychological distress (the Hospital Depression and Anxiety Scale). All outcomes were measured at baseline, 3 days, 1 week, 2 weeks, and 3 weeks.

**Results:**

The pain intensity in both groups decreased gradually from day 3 and decreased to the lowest at the week 3, with a decrease rate of 41.09% in the rTMS group and 23.23% in the sham group. The NRS score of the rTMS group was significantly lower than that of the sham group on the week 2 (*p* < 0.001, Cohen’s d =1.135) and week 3 (*p*=0.017, Cohen’s d = -0.822). The OME daily dose, physiology and psychology domains of WHOQOL-BREF scores, as well as the HAM-A and HAM-D scores all were significantly improved at week 3 in rTMS group.

**Conclusion:**

Advanced NSCL patients with cancer pain treated with rTMS showed better greater pain relief, lower dosage of opioid, and better mood states and quality of life. rTMS is expected to be a new effective adjuvant therapy for cancer pain in advanced NSCLC patients.

## Introduction

Recently, the incidence of cancer in the world is increasing year by year (1). According to the studies, at least 25-30% of newly diagnosed cancer patients are associated with pain, and the incidence of pain in patients with advanced cancer is as high as 74% (2, 3). Lung cancer is the first cause of cancer death in the world and it is mostly diagnosed at an advanced stage (4). Lung cancer-related pain mainly depends on the location of the primary tumor, local infiltration of the tumor, visceral and lymph node metastasis, compression of nerve and bone metastasis, etc. Pain is a complex symptom that affects many aspects of a cancer patient, including physical function, sleep, ability of daily living, psychological and emotional status, and social relations (5). Therefore, intervention on the pain is of great significance to improve the quality of life and the prognosis of lung cancer patients (6).

The WHO three-step ladder for cancer pain, which is widely used in clinic, follows the rule that the choice of analgesic drugs were based on the pain intensity: step I-nonsteroidal anti-inflammatory drugs (eg, aspirin or ibuprofen) to mild pain, step II- weak opioids (eg, codeine or tramadol) to moderate pain, and step III- strong opioids (morphine or oxycodone) to severe pain (7). Meanwhile, there were also some other treatments for cancer pain, such as radiotherapy, surgery, chemotherapy, radioisotope therapy, bisphosphonate and so on (8). Although those treatments effectively relieve the symptoms of cancer patients to some extent, there were still 50% patients whose pain is undertreated. The serious pain affects the quality of life of cancer patients (9).

In the past two decades, neuromodulation technique has gradually become a new direction of pain treatment. Repetitive transcranial magnetic stimulation (rTMS) is one of the most commonly used non-invasive neuromodulation techniques in clinic (10). rTMS could produce a certain intensity of magnetic field by focusing on the brain with a specific shape coil, which makes cortex neuron depolarization or hyperpolarization, and then regulate the excitability of neuron. Usually, low frequency (< 1Hz) stimulation has an inhibitory effect on the brain, while high frequency (> 5Hz) stimulation excite neurons (11). It has been reported that rTMS relieves various types of pain, such as neuropathic pain after spinal cord injury, stroke or postoperative of trigeminal nerve (12), migraine (13), fibromyalgia (14) and chronic musculoskeletal pain (15). Even less clinical study has been done on the application of rTMS in patients with cancer pain.

In addition, patients with cancer pain are often complicated with depression, which aggravates the complexity and difficulty of cancer pain treatment, and that is also one of the main reasons for poor analgesic effect (16). Many studies have found that high frequency rTMS stimulation of the dorsolateral prefrontal cortex (DLPFC) is an effective method for the treatment of depression (17). At the same time, high-frequency stimulation also relieves neuropathic pain and chronic pain to a certain extent (18). Until now, Food and Drug Administration (FDA) in the United States has officially approved the use of rTMS in the treatment of depression and migraine (19, 20). Based on the above evidence, we hypothesize that high frequency rTMS stimulation in the DLPFC may be a new and effective treatment for cancer pain. Given the efficacy of rTMS on cancer pain remains unclear, we therefore conducted a randomized controlled trial to firstly explore the analgesic effect of rTMS on advanced non-small-cell lung cancer (NSCLC) patients.

## Methods

### Study Design

This trial was a randomized, double-blind, parallel-group, sham-controlled clinical trial, approved by the Ethics Committee of Chongqing Cancer Hospital and was registered (ChiCTR.org.cn identifier: ChiCTR 2000029130). All patients provided written informed consents before enrolling. After completing consent forms, patients were randomly assigned to either the rTMS group or the sham stimulation group using a block randomization scheme. An independent investigator carried out the randomization. Two independent, trained assessors and enrolled patients were blinded to the group assignments.

### Participants

NSCLC patients with cancer pain were enrolled consecutively from January 2020 to March 2021. The inclusion criteria were: 1) confirmed diagnosis of advanced NSCLC by pathology or cytology, 2) accompanied with pain symptoms, and confirmed as cancer pain by oncologist, 3) experienced worst pain score≥4 (0-to-10 numeric rating scale [NRS]) at the site of pain, 4) age between 18 and 70 years, 5) with clear awareness, and could cooperate to evaluate pain severity, 6) estimated that the survival time is more than 3 months, 7) with completion of signed informed consents, and voluntary participation in this study. The exclusion criteria were: 1) brain tumor patients 2) history of seizure, 3) implanted pacemaker, stent and other metal substances 4) acute pain anywhere in the body due to other diseases, 5) serious psychiatric diagnoses (eg, psychosis)

### Interventions

#### Analgesia Treatment

All the participants were treated with medications according to WHO three-step principle. In order to facilitate the statistics of the dosage of opioids in patients with cancer pain, morphine sulfate controlled-release tablets or oxycodone hydrochloride sustained-release tablets were used for analgesia in this study (21).

#### rTMS Procedure

rTMS was applied with a magnetic stimulator (CCY-I) with a figure-of-eight coil (B9076; 22 mm inner diameter, 90 mm outer diameter, 76 mm combined long axis length, Wuhan Yiruide Medical Equipment, Wuhan, China). The rTMS protocols used in this study were in accordance with the safety guidelines for rTMS applications (22). The resting motor threshold (RMT) was measured as follows (22): a single TMS pulse stimulated one side of the primary motor cortex, the motor evoked potential (MEP) was recorded at the first dorsal interosseous (FDI) muscle of the contralateral hand with a surface electrode. The RMT was defined as the lowest stimulation intensity capable of eliciting MEPs ≥ 50μV peak-to-peak amplitude in at least five of ten consecutive stimulations (23). Parameter setting were as follows (23): stimulation target, left side DLPFC; stimulation intensity, 80% RMT; frequency, 10HZ; 15 pulse trains (1.5 s), with intertrain intervals of 3 s (total of 1500pulses). In the control group, the sham stimulation was delivered using a same coil, but with no magnetic stimulation output (only emitting the same sound, with different stimulation angles). Both the two groups (rTMS group and control group) received stimulation once a day, 5 days per week, for a total of 3 weeks.

### Outcome Measurements

**Figure 1:**
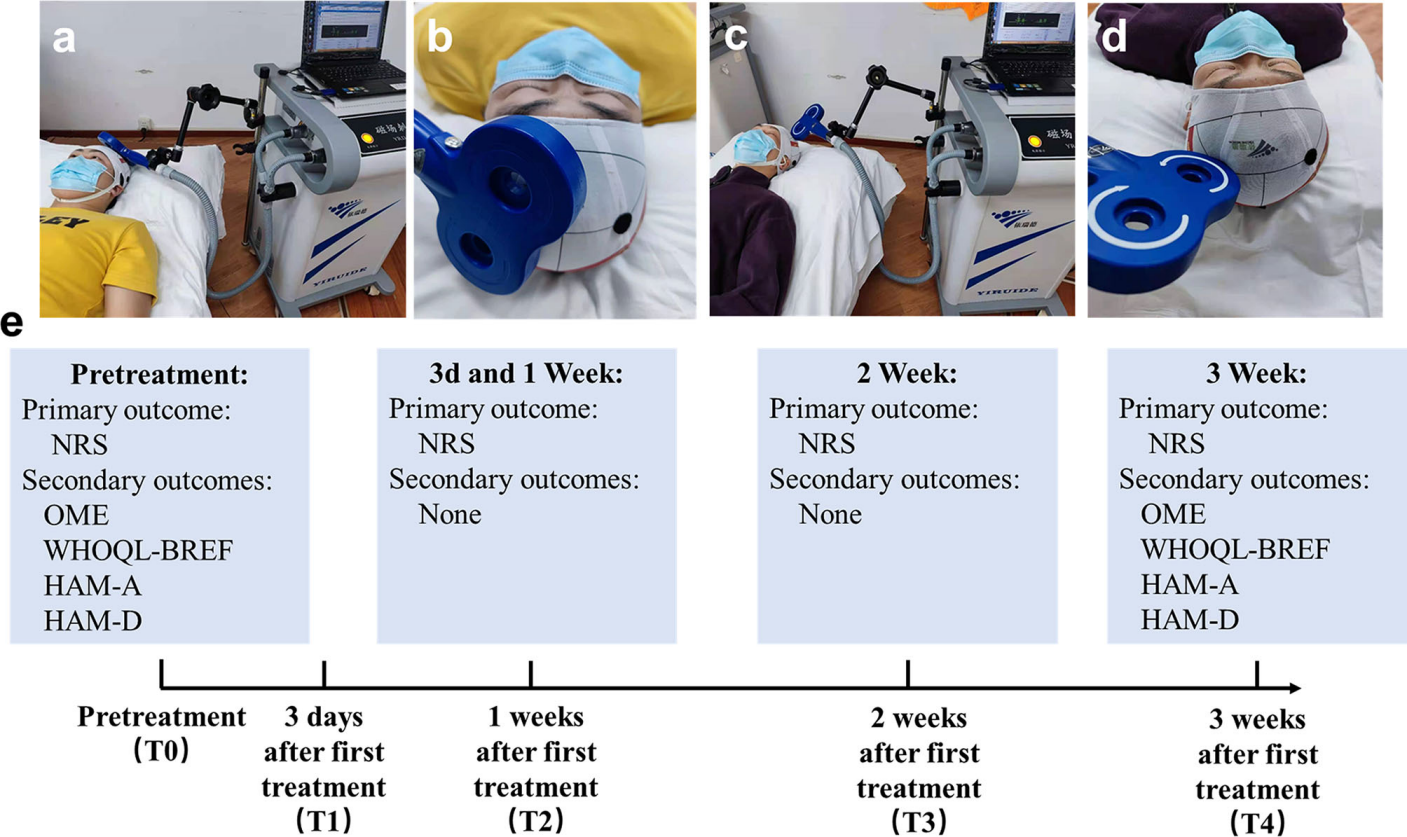
Schematic diagram of the experimental design. **(A)** Image of the treatment in rTMS group, **(B)** Close-up of the coil in rTMS group. **(C)** Image of the treatment in sham group, **(D)** Close-up of the coil in sham group, **(E)** Primary and secondary outcomes at different time points.

The pain intensity was assessed at 1 day before rTMS treatment (T0), 3 days (T1), 1 week (T2), 2 weeks (T3) and 3 weeks (T4) after first rTMS treatment. Mood status and quality of life were evaluated only at T0 and T4 timepoints. **Figure 1** shows the research and evaluation schedule. The primary outcome was:

1. NRS. The NRS consists of 11-point scale, of which 0 represents no pain and 10 represents the strongest pain imaginable, which has been recommended by the Initiative on Methods, Measurement, and Pain Assessment in Clinical Trials (IMMPACT) guidelines (24).

The secondary outcomes were as follows:

1. Oral morphine equivalent (OME). The conversion method of OME refer to the previous literature reports: oral oxycodone hydrochloride is 1:1.5, intravenous morphine is 1:3 converted to oral morphine (25).
2. WHO Quality of Life-BREF (WHOQL-BREF) (26). Quality of life was evaluated by means of the WHOQOL-BREF. The WHOQOL-BREF generates a profile and score for each of the 4 domains, including physiology, psychology, social relationship and environment. Each domain score ranges 0-100, higher scores represent better quality of life.
3. Hamilton Anxiety Scale (HAM-A) (27). HAM-A was used to evaluate the severity of anxiety. HAM-A includes 14 items; each item score ranges 0-4, higher scores represent more severe anxiety.
4. Hamilton Depression Scale (HAM-D) (28). HAM-D was used to evaluate the severity of depression. HAM-D includes 17 items; each item score ranges 0-4, higher scores represent more severe depression.

### Statistical Analysis

Sample size was estimated using the G Power v.3.1 statistical tool. To achieve a statistical power of 85% with statistical significance at *P*<0.05 (two-sided test) and an effect size of *r* = 0.45, a total minimum sample size of 38 patients was required. Considering an estimated 10% dropout rate, the sample size was inflated to 21 participants per group (N = 42).

Statistical data analyses were performed using IBM SPSS (version 21; SPSS, Chicago, IL). Baseline group differences were explored with *t* tests or chi-squared tests. A repeated-measures analysis of variance (rmANOVA) was used to analyze the data for the efficacy of rTMS, with time as the within-subjects factor and treatment as the between-subjects factor. *Post hoc* analyses were performed with Bonferroni adjustment for further multiple comparisons. *P*<0.05 were considered statistically significant. The percentage of change within each individual was calculated as follows: [(post-treatment - pre-treatment score)/(pre-treatment score)] *100.

## Results

**Figure 2:**
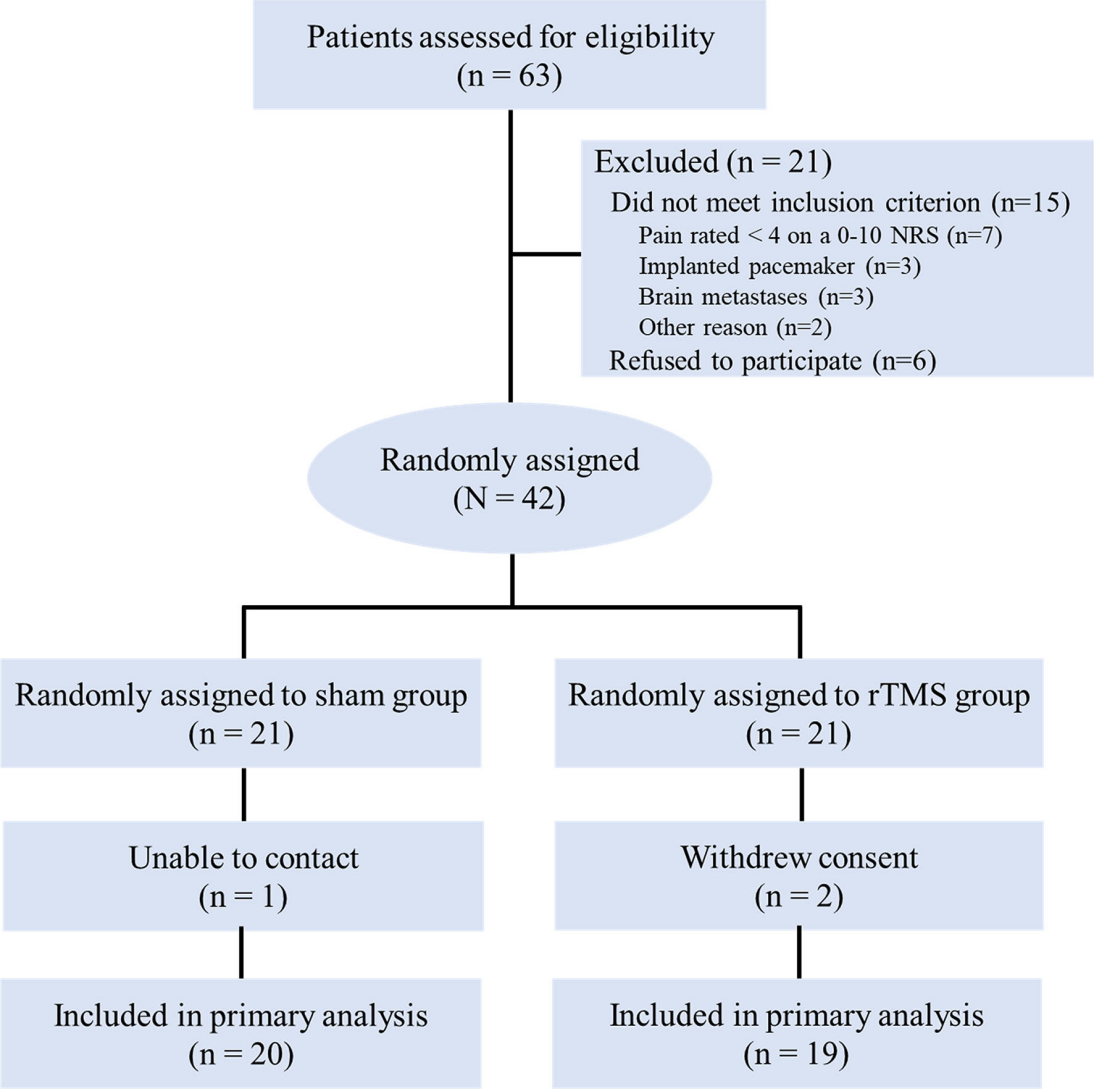
CONSORT diagram. NRS, numeric rating scale; rTMS, repetitive transcranial magnetic stimulation.

A total of 63 advanced NSCLC patients with cancer pain were screened and 42 eligible patients were enrolled in the study (**Figure 2**). They were randomly allocated into the rTMS group (n = 21) or the sham group (n = 21). One case in the rTMS group withdrew from the study because of moving to another city in the second week, while two cases in the sham group withdrew from the study because of unwilling to continue to participate in the study in the first week. The remaining 39 patients completed the 3-week trial, with 20 cases in the rTMS group and 19 cases in the sham group. There were no significant differences between the two groups in terms of demographic variables or clinical characteristics (**Table 1**). Of the 39 patients, two patients in the rTMS group reported transient scalp numbness or facial muscle twitching during the rTMS therapy, but no serious adverse effects were observed.

**Table 1:** Baseline Demographics and Clinical Characteristics by Group.

Patient Demographics and Characteristics	rTMS group (n = 20)	sham group (n = 19)	*P* value
Demographics			
Female/Male	8/12	9/10	0.643
Age, mean ± SD, years	58.5 ± 8.9	59.6 ± 7.7	0.669
>60 years	9 (45.0%)	10 (50.0%)	0.633
BMI, mean ± SD, kg/m^2^	21.4 ± 1.9	21.0 ± 1.5	0.505
Clinical characteristics			
Pathological type			0.72
Adenocarcinoma	13 (65.0%)	10 (52.6%)	
Squamous cell carcinoma	5 (25.0%)	6 (31.6%)	
Others	2 (5%)	3 (15.8%)	
neoplasm stage			0.946
III B	3 (15.0%)	3 (15.8%)	
IV	17 (85.0%)	16 (84.2%)	
ECOG performance status			0.839
0-1	12 (60.0%)	12 (63.2%)	
≥2	8 (40.0%)	7 (36.8%)	
Number of organ metastasis			0.557
0–2	13 (65.0%)	14 (73.7%)	
≥3	7 (35.0%)	5 (26.3%)	
Current antitumor treatment	12 (60%)	11 (57.9%)	0.893
Pain site			0.811
Bone pain	9 (45.0%)	10 (52.7%)	
Chest pain	9 (45.0%)	8 (42.1%)	
Other	2 (10%)	1 (5.2%)	
Pain at baseline, mean ± SD	6.5 ± 1.7	6.4 ± 1.6	0.882
OME daily dose, mean ± SD, mg	109.5 ± 52.5	115.8 ± 59.6	0.735

Data are presented as No. (%) unless indicated otherwise.

BMI, body mass index; ECOG, Eastern Cooperative Oncology Group; OME, oral morphine equivalents; SD, standard deviation.

### Primary Outcome

#### Pain Scores

**Figure 3:**
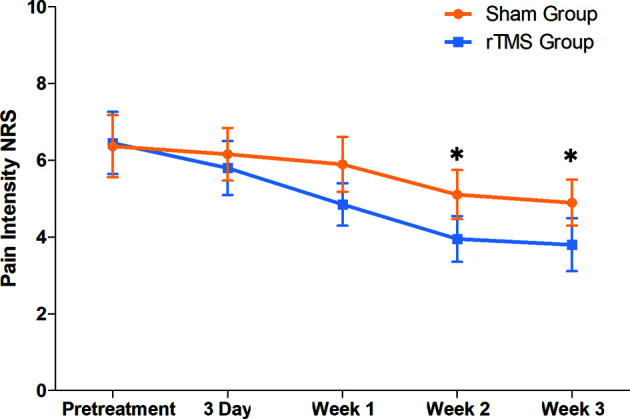
Raw scores on primary outcome (NRS) from baseline to 3 weeks. The NRS scores were calculated by the average for the preceding 7 days. Error bars indicate 95% confidence intervals, * indicate significant inter-group difference, *P* < 0.05. NRS, numeric rating scale.

The pain intensity before treatment was 6.45 (SD,1.69) in the rTMS group and 6.37 (SD,1.63) in sham group. The pain intensity in both groups decreased gradually from day 3 and decreased to the lowest at the week 3, with a decrease of 41.09% in the rTMS group and 23.23% in the sham group. The NRS score for the rTMS group was significantly lower than that of the sham group on the week 2 (*P* = 0.04, Cohen’s d =-0.735) and week 3 (*P* = 0.017, Cohen’s d =- 0.822) (**Table 2** and **Figure 3**).

### Secondary Outcome

#### Oral Morphine Equivalent (OME)

**Figure 4:**
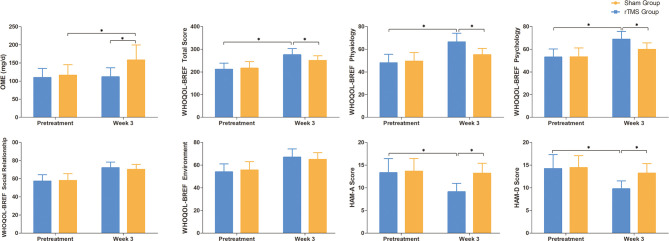
Raw scores on secondary outcomes at baseline and weeks 3. Error bars indicate 95% confidence intervals, * indicate significant inter-group or pre-post differences, *P* < 0.05. OME, Oral morphine equivalent; WHOQL-BREF, WHO Quality of Life-BREF; HAM-A, Hamilton Anxiety Scale; HAM-D, Hamilton Depression Scale.

The OME in the rTMS group at baseline and week 3 were 109.5 ± 52.5mg and 111.5 ± 52.4, respectively, and those in the sham group were 115.8 ± 59.6mg and 157.9 ± 84.3 mg, respectively. On week 3, the OME in the rTMS group was similar to that of baseline (*P* = 0.02, Cohen’s *d* = 0.796), while the sham group both were significantly higher than that of baseline (*P* = 0.02, Cohen’s *d* = 0.796) (**Table 2** and **Figure 4**).

**Table 2:** Primary and Secondary Outcomes.

Outcome	rTMS group (n = 20)			sham group (n = 19)			*Cohen’s d (rTMS to sham at 3 weeks)*	*P* value
	Baseline	3 Weeks	Change From Baseline	Baseline	3 Weeks	Change From Baseline		
**Primary outcome**								
NRS	6.5 (1.7)	3.8 (1.4)	-2.7 (1.2)	6.4 (1.6)	4.9 (1.2)	-1.5 (1.3)	-0.822	0.017
**Secondary Outcome**								
OME daily dose, mg	109.5(52.5)	111.5(52.5)	2.0 (12.5)	111.8(59.7)	157.9(84.3)	42.1 (31.7)	-0.603	0.05
WHOQOL-BREF domain								
Physiology	47.9 (15.9)	66.3 (16.3)	15.8 (13.9)	49.4 (15.5)	55.1 (11.4)	8.3 (9.5)	0.796	0.02
Psychology	52.4 (14.3)	69.7 (14.9)	12.6 (7.4)	53.2 (16.1)	59.7 (11.7)	8.1 (5.5)	0.746	0.031
Social relationship	57.1 (15.1)	72.0 (13.1)	12.4 (10.2)	57.8 (15.5)	70.1 (11.4)	10.9 (2.0)	0.155	0.638
Environment	53.9 (14.9)	66.9 (15.1)	11.8 (8.9)	55.5 (14.9)	64.9 (12.2)	8.9 (10.6)	0.146	0.654
HAM-A	13.3 (6.5)	9.1 (3.9)	-4.2(3.5)	13.6 (5.7)	13.2 (4.5)	-0.5 (2.8)	-0.949	0.005
HAM-D	14.1 (5.9)	9.9 (3.6)	-4.2(2.9)	14.3 (5.4)	13.2 (4.2)	-1.1(2.1)	-0.869	0.011

Data given as mean (SD) NRS, numeric rating scale; OME, oral morphine equivalent; SD, standard deviation; WHOQOL-BREF, World Health Organization Quality of Life-BREF; HAM-A, Hamilton Anxiety Scale; HAM-D, Hamilton Depression Scale.

### Quality of Life

There were significant improvements in all domains of WHOQOL-BREF scores for both the groups when compared with baseline after 3 weeks of treatment. The physiology and psychology domains of WHOQOL-BREF scores showed significant improvements with rTMS group versus sham group (*P* = 0.02, Cohen’s *d* = 0.796 and *P* = 0.031, Cohen’s *d* = 0.746, respectively). (**Table 2** and **Figure 4**).

### Mood Changes: HAM-A and HAM-D

The HAM-A and HAMD scores in the rTMS group showed significant improvements after 3 weeks of treatment when compared with baseline (*P* = 0.005, Cohen’s *d* = -0.949 and *P* = 0.011, Cohen’s *d* =- 0.869, respectively). However, there were no significant improvements in the sham group (**Table 2** and **Figure 4**).

## Discussion

The findings of this randomized, double-blind, sham-controlled trial showed a significant analgesic benefit by using rTMS in advanced NSCLC patients with cancer pain. Moreover, our study also showed rTMS could reduce the daily dosage of opioids and improve the quality of life and psychological distress of NSCLC patients with cancer pain. To our knowledge, this is the first randomized controlled trial to explore the effective analgesic treatment of rTMS in patients with cancer pain. Accordingly, we expect our findings could have clinical implications.

Recently, there has been increasing evidence that rTMS is a noninvasive and safe treatment option for pain that may benefit patients who do not respond to conventional pharmacological therapies (29). Functional imaging studies have shown that there was a cognitive regulation circuit of pain in the brain (30). When the pain information is transmitted upward from the spinal cord to the brain, it enters the thalamus, amygdala, anterior cingulate gyrus, primary/secondary somatosensory cortex and other brain areas, and forms the pain sensation or pain emotion through the structural and functional connection with prefrontal cortex. DLPFC directly promotes or inhibits pain through coordination with these brain regions or through modulating the activity of pain descending inhibition pathways (31). Therefore, DLPFC-rTMS was able to reduce pain sensation, as supported by several recent studies, like spinal cord injury (12), migraine (13) and fibromyalgia (14). In this study, we hypothesized that DLPFC-rTMS may also be effective in the treatment of cancer pain and thus conducted this trial. In our trial, the pain intensity in the rTMS group at week 3 was decreased by 2.7 points from baseline, which was significantly higher than the 1.5 points in the sham group. The pain reduction of 2.7 points in the rTMS group exceeded the 2-point reduction by using NRS scale that has been recommended as a clinically significant improvement by the IMMPACT study (24). Meanwhile, we also found that DLPFC-rTMS can reduce the dosage of opioids in NSCLC patients with cancer pain. Reduced pain intensity, together with less opioid dose, revealed the clinical benefit of DLPFC-rTMS in the treatment of cancer pain in NSCLC patients.

In addition to pain, it is evident that mood disorders are also a major problem for NSCLC patients. Undertreatment of cancer pain is often accompanied by physical fitness decline, fatigue and sleep disorders, and even anxiety and depression, which obviously increase the difficulty of analgesic treatment and reduce the quality of life for patients (9). Our data on anxiety and depression, from HAMA and HAMD also demonstrated the existence of poor psychological status among NSCLC patients with cancer pain, which was consistent with the previous studies (32, 33). Therefore, we should pay special attention to the treatment of psychological disorders in patients with cancer pain (16, 34). As shown in this study, improvements in anxiety and depression were significant higher in the rTMS group than that of in the sham group (P<0.05). We have also found that DLPFC-rTMS treatment could significantly improve the quality of life versus sham stimulation. This finding is consistent with the role of the DLPFC in modulating brain regions involved in emotions such as the anterior cingulate cortex and insular cortex (35, 36). DLPFC-rTMS has been approved in the US FDA to treat major depressive disorder in adults who have not responded to prior antidepressant medications (19), and this effect might account for the improvement of the mood disorders in patients who were treated with DLPFC-rTMS in this study.

There are some strengths in this study. We provided the first clinical RCT study in which high frequency rTMS over the DLPFC is able to decrease cancer pain in advanced NSCLC patients. The undertreatment of cancer pain is still very common in clinic. Clinicians usually gradually increase the dose of analgesic medications. If the findings of this study were further confirmed by multicenter, large sample clinical trials, rTMS could be used clinically as a convenient and effective non-drug adjuvant therapeutic tool. Meanwhile, we also evaluated the outcome comprehensively and appropriately. The outcome measures in our study were consistent with IMMPACT recommendations for chronic pain (24, 37), including measures of dosage of opioids, pain intensity, physical functioning, mood status and quality of life, which ensured a comprehensive evaluation of the treatment. However, there are also some limitations in this study. First, the relatively small number of participants and a single-center trial design is the main limitation of our study, which may increase the risk of type II error. Future large-scale multicenter studies are needed to ameliorate this limitation. Second, due to the small sample size, we didn’t conduct further subgroup analysis to explore the differences in the efficacy of rTMS in patients with different stages of metastasis. Third, it is unknown whether the analgesic effects of rTMS on patients with cancer pain are long-lasting, due to lack of long-term follow-up. Fourth, the effects of rTMS on patients’ sleep quality and the cost-effective analysis of rTMS on pain treatment are also very interesting questions, future studies could further explore them.

In conclusion, our results support the use of rTMS as a promising adjuvant therapeutic tool for cancer pain, with its dual beneficial effect in decreasing pain intensity and psychological distress in advanced NSCLC patients. If this effect can be confirmed in future larger sample studies, it will undoubtedly have a better clinical application prospect for patients with cancer pain.

## Data Availability Statement

The raw data supporting the conclusions of this article will be made available by the authors, without undue reservation.

## Ethics Statement

The studies involving human participants were reviewed and approved by Ethics Committee of Chongqing Cancer Hospital. The patients/participants provided their written informed consent to participate in this study. Written informed consent was obtained from the individual(s) for the publication of any potentially identifiable images or data included in this article.

## Author Contributions

YT and Y-sL conceived and designed the study. YT and HC analyzed the experimental data and drafted the manuscript. YT, M-lT, and YZ collected the experimental data. Y-sL, S-lX, YL and X-hJ revised the manuscript for important intellectual content. All authors contributed to the article and approved the submitted version.

## Funding

This study was supported by the National Nature Science Foundation of China (81902302) and Chongqing Natural Science Foundation (cstc2018jcyjAX0772).

## Conflict of Interest

The authors declare that the research was conducted in the absence of any commercial or financial relationships that could be construed as a potential conflict of interest.

## Publisher’s Note

All claims expressed in this article are solely those of the authors and do not necessarily represent those of their affiliated organizations, or those of the publisher, the editors and the reviewers. Any product that may be evaluated in this article, or claim that may be made by its manufacturer, is not guaranteed or endorsed by the publisher.

## References

[1] Mao JJ Pillai GG Andrade CJ Ligibel JA Basu P Cohen L. Integrative Oncology: Addressing the Global Challenges of Cancer Prevention and Treatment. CA Cancer J Clin (2022) 72(2):144–64. doi: 10.3322/caac.21706 PMC13183357 34751943

[2] Neufeld NJ Elnahal SM Alvarez RH. Cancer Pain: A Review of Epidemiology, Clinical Quality and Value Impact. Future Oncol (2017) 13(9):833–41. doi: 10.2217/fon-2016-0423 27875910

[3] Goudas LC Bloch R Gialeli-Goudas M Lau J Carr DB. The Epidemiology of Cancer Pain. Cancer Invest (2005) 23(2):182–90. doi: 10.1081/CNV-200050482 15813511

[4] Wu LL Li CW Lin WK Qiu LH Xie D. Incidence and Survival Analyses for Occult Lung Cancer Between 2004 and 2015: A Population-Based Study. BMC Cancer (2021) 21(1):1009. doi: 10.1186/s12885-021-08741-4 34496775 PMC8427887

[5] Mantyh PW. Cancer Pain and its Impact on Diagnosis, Survival and Quality of Life. Nat Rev Neurosci (2006) 7(10):797–809. doi: 10.1038/nrn1914 16988655

[6] Wilton J Abdia Y Chong M Karim ME Wong S MacInnes A. Prescription Opioid Treatment for non-Cancer Pain and Initiation of Injection Drug Use: Large Retrospective Cohort Study. BMJ (2021) 375:e066965. doi: 10.1136/bmj-2021-066965 34794949 PMC8600402

[7] WHO. World Health Organization Cancer Pain Relief, With a Guide to Opioid Availability. ed 2. Geneva, Switzerland: World Health Organization (1996).

[8] Fallon M Giusti R Aielli F Hoskin P Rolke R Sharma M. Management of Cancer Pain in Adult Patients: ESMO Clinical Practice Guidelines. Ann Oncol (2018) 29(Suppl 4):iv166–iv91. doi: 10.1093/annonc/mdy152 30052758

[9] Deandrea S Montanari M Moja L Apolone G. Prevalence of Undertreatment in Cancer Pain. A Review of Published Literature. Ann Oncol (2008) 19(12):1985–91. doi: 10.1093/annonc/mdn419 PMC2733110 18632721

[10] Jiang X Yan W Wan R Lin Y Zhu X Song G. Effects of Repetitive Transcranial Magnetic Stimulation on Neuropathic Pain: A Systematic Review and Meta-Analysis. Neurosci Biobehav Rev (2022) 132(1):130–41. doi: 10.1016/j.neubiorev.2021.11.037 34826512

[11] Edemann-Callesen H Winter C Hadar R. Using Cortical non-Invasive Neuromodulation as a Potential Preventive Treatment in Schizophrenia - A Review. Brain Stimul (2021) 14(3):643–51. doi: 10.1016/j.brs.2021.03.018 33819680

[12] Zhang KL Yuan H Wu FF Pu XY Liu BZ Li Z. Analgesic Effect of Noninvasive Brain Stimulation for Neuropathic Pain Patients: A Systematic Review. Pain Ther (2021) 10(1):315–32. doi: 10.1007/s40122-021-00252-1 PMC8119533 33751453

[13] Ornello R Caponnetto V Ratti S D'Aurizio G Rosignoli C Pistoia F. Which is the Best Transcranial Direct Current Stimulation Protocol for Migraine Prevention? A Systematic Review and Critical Appraisal of Randomized Controlled Trials. J Headache Pain (2021) 22(1):144. doi: 10.1186/s10194-021-01361-0 34837963 PMC8903540

[14] Su YC Guo YH Hsieh PC Lin YC. Efficacy of Repetitive Transcranial Magnetic Stimulation in Fibromyalgia: A Systematic Review and Meta-Analysis of Randomized Controlled Trials. J Clin Med (2021) 10(20):4669. doi: 10.3390/jcm10204669 34682790 PMC8538417

[15] Kandic M Moliadze V Andoh J Flor H Nees F. Brain Circuits Involved in the Development of Chronic Musculoskeletal Pain: Evidence From Non-Invasive Brain Stimulation. Front Neurol (2021) 12:732034. doi: 10.3389/fneur.2021.732034 34531819 PMC8438114

[16] Lyman GH Greenlee H Bohlke K Bao T DeMichele AM Deng GE. Integrative Therapies During and After Breast Cancer Treatment: ASCO Endorsement of the SIO Clinical Practice Guideline. J Clin Oncol (2018) 36(25):2647–55. doi: 10.1200/JCO.2018.79.2721 PMC13123314 29889605

[17] Cappon D den Boer T Jordan C Yu W Metzger E Pascual-Leone A. Transcranial Magnetic Stimulation (TMS) for Geriatric Depression. Ageing Res Rev (2022) 74(2):101531. doi: 10.1016/j.arr.2021.101531 34839043 PMC8996329

[18] Che X Cash RFH Luo X Luo H Lu X Xu F. High-Frequency rTMS Over the Dorsolateral Prefrontal Cortex on Chronic and Provoked Pain: A Systematic Review and Meta-Analysis. Brain Stimul (2021) 14(5):1135–46. doi: 10.1016/j.brs.2021.07.004 34280583

[19] Perera T George MS Grammer G Janicak PG Pascual-Leone A Wirecki TS. The Clinical TMS Society Consensus Review and Treatment Recommendations for TMS Therapy for Major Depressive Disorder. Brain Stimul (2016) 9(3):336–46. doi: 10.1016/j.brs.2016.03.010 PMC5612370 27090022

[20] Clark O Mahjoub A Osman N Surmava AM Jan S Lagman-Bartolome AM. Non-Invasive Neuromodulation in the Acute Treatment of Migraine: A Systematic Review and Meta-Analysis of Randomized Controlled Trials. Neurol Sci (2022) 43(1):153–65. doi: 10.1007/s10072-021-05664-7 34698941

[21] Bandieri E Romero M Ripamonti CI Artioli F Sichetti D Fanizza C. Randomized Trial of Low-Dose Morphine Versus Weak Opioids in Moderate Cancer Pain. J Clin Oncol (2016) 34(5):436–42. doi: 10.1200/JCO.2015.61.0733 26644526

[22] Rossi S Hallett M Rossini PM Pascual-Leone A. And Safety of TMSCG. Safety, Ethical Considerations, and Application Guidelines for the Use of Transcranial Magnetic Stimulation in Clinical Practice and Research. Clin Neurophysiol (2009) 120(12):2008–39. doi: 10.1016/j.clinph.2009.08.016 PMC3260536 19833552

[23] Zhao CG Sun W Ju F Jiang S Wang H Sun XL. Analgesic Effects of Navigated Repetitive Transcranial Magnetic Stimulation in Patients With Acute Central Poststroke Pain. Pain Ther (2021) 10(2):1085–100. doi: 10.1007/s40122-021-00261-0 PMC8586137 33866522

[24] Dworkin RH Turk DC Farrar JT Haythornthwaite JA Jensen MP Katz NP. Core Outcome Measures for Chronic Pain Clinical Trials: IMMPACT Recommendations. Pain (2005) 113(1-2):9–19. doi: 10.1016/j.pain.2004.09.012 15621359

[25] Hale M Wild J Reddy J Yamada T Arjona Ferreira JC. Naldemedine Versus Placebo for Opioid-Induced Constipation (COMPOSE-1 and COMPOSE-2): Two Multicentre, Phase 3, Double-Blind, Randomised, Parallel-Group Trials. Lancet Gastroenterol Hepatol (2017) 2(8):555–64. doi: 10.1016/S2468-1253(17)30105-X 28576452

[26] Xia P Li N Hau KT Liu C Lu Y. Quality of Life of Chinese Urban Community Residents: A Psychometric Study of the Mainland Chinese Version of the WHOQOL-BREF. BMC Med Res Methodol (2012) 12:37. doi: 10.1186/1471-2288-12-37 22452994 PMC3364902

[27] Hamilton M. The Assessment of Anxiety States by Rating. Br J Med Psychol (1959) 32(1):50–5. doi: 10.1111/j.2044-8341.1959.tb00467.x 13638508

[28] Hamilton M. A Rating Scale for Depression. J Neurol Neurosurg Psychiatry (1960) 23:56–62. doi: 10.1136/jnnp.23.1.56 14399272 PMC495331

[29] Hosomi K Sugiyama K Nakamura Y Shimokawa T Oshino S Goto Y. A Randomized Controlled Trial of 5 Daily Sessions and Continuous Trial of 4 Weekly Sessions of Repetitive Transcranial Magnetic Stimulation for Neuropathic Pain. Pain (2020) 161(2):351–60. doi: 10.1097/j.pain.0000000000001712 PMC6970577 31593002

[30] Tracey I Mantyh PW. The Cerebral Signature for Pain Perception and its Modulation. Neuron (2007) 55(3):377–91. doi: 10.1016/j.neuron.2007.07.012 17678852

[31] Martin L Borckardt JJ Reeves ST Frohman H Beam W Nahas Z. A Pilot Functional MRI Study of the Effects of Prefrontal rTMS on Pain Perception. Pain Med (2013) 14(7):999–1009. doi: 10.1111/pme.12129 23647651

[32] Johannsen M O'Connor M O'Toole MS Jensen AB Hojris I Zachariae R. Efficacy of Mindfulness-Based Cognitive Therapy on Late Post-Treatment Pain in Women Treated for Primary Breast Cancer: A Randomized Controlled Trial. J Clin Oncol (2016) 34(28):3390–9. doi: 10.1200/JCO.2015.65.0770 27325850

[33] Bradt J Dileo C Myers-Coffman K Biondo J. Music Interventions for Improving Psychological and Physical Outcomes in People With Cancer. Cochrane Database Syst Rev (2021) 10:CD006911. doi: 10.1002/14651858.CD006911.pub4 34637527 PMC8510511

[34] Greenlee H DuPont-Reyes MJ Balneaves LG Carlson LE Cohen MR Deng G. Clinical Practice Guidelines on the Evidence-Based Use of Integrative Therapies During and After Breast Cancer Treatment. CA Cancer J Clin (2017) 67(3):194–232. doi: 10.3322/caac.21397 28436999 PMC5892208

[35] Che X Cash R Chung SW Bailey N Fitzgerald PB Fitzgibbon BM. The Dorsomedial Prefrontal Cortex as a Flexible Hub Mediating Behavioral as Well as Local and Distributed Neural Effects of Social Support Context on Pain: A Theta Burst Stimulation and TMS-EEG Study. Neuroimage (2019) 201:116053. doi: 10.1016/j.neuroimage.2019.116053 31351163

[36] Ferenczi EA Zalocusky KA Liston C Grosenick L Warden MR Amatya D. Prefrontal Cortical Regulation of Brainwide Circuit Dynamics and Reward-Related Behavior. Science (2016) 351(6268):aac9698. doi: 10.1126/science.aac9698 26722001 PMC4772156

[37] Jiang J Li Y Shen Q Rong X Huang X Li H. Effect of Pregabalin on Radiotherapy-Related Neuropathic Pain in Patients With Head and Neck Cancer: A Randomized Controlled Trial. J Clin Oncol (2019) 37(2):135–43. doi: 10.1200/JCO.18.00896 30457920

